# Competition and Predation in Soil Fungivorous Microarthropods Using Stable Isotope Ratio Mass Spectrometry

**DOI:** 10.3389/fmicb.2019.01274

**Published:** 2019-06-07

**Authors:** Felicity V. Crotty, Sina M. Adl

**Affiliations:** Department of Soil Science, University of Saskatchewan, Saskatoon, SK, Canada

**Keywords:** fungivory, microarthropods, nutrient cycling, stable isotopes, trophic interactions

## Abstract

The soil food web is often described as having three main energy channels: root, bacterial and fungal. Here we provide quantitative data using a sensitive stable isotope ratio mass spectrometry procedure with microcosms on species interactions in the fungal pathway. We measured ^15^N and ^13^C enrichment in microarthropods through grazing rare isotope enriched fungal mycelia. Experimental treatments were various combinations of 1, 2, 3, 4 microarthropods species. We used three fungivores (the collembolan *Lepidocyrtus curvicollis*, the Astigmata *Tyrophagus putrescentiae*, the Oribatida *Oribatula tibialis*), and the Mesostigmata predator *Hypoaspis acquilifer.* We collected individuals of each species separately, as well as their feces, and molt where available. All three fungivorous microarthropods consumed significantly more than their own body weight per day. The three fungivores differed in their consumption of the mycelium as it was not equally palatable to each. The Mesostigmata predator *Hypoaspis* also differed in its microarthropod prey preference. In multiple species combinations microarthropod behavioral interactions modified consumption and predation rates. Our selection of mites of different sizes, with varied preference for the mycelium, combined with differing predation rates on each mite, demonstrate that even three trophic level interactions with only five interacting species are not predictably simple. The interpretation of the stable isotope results and consumed-excreted weights indicate that: (a) behavior and microscopic observations should not be ignored in competition-predation interactions, and (b) functional guilds can take advantage of more diverse food opportunities. The reality of mixed diets complicates functional guild assignments that are reflected in ^15^N and ^13^C isotope levels at natural abundances in the environment. Microcosm experiments with this sensitive technique can help decipher the interpretation of rare isotope natural abundance values, as well as providing measured consumption, growth, and excretion rate values for modeling soil food web interactions.

## Introduction

Soil food web models typically recognize three main routes energy can flow through the soil – roots, the fungal or bacterial pathway (Moore et al., 2005). An early study of soil food web structure found that both top-down control and bottom-up control existed simultaneously and were necessary for ecosystem stability (de Ruiter et al., 1995). Increased predation rate on prey, or of grazing consumption (e.g., on bacteria lawn, a mycelium, or pasture) decreases the biomass and abundance of the species consumed, while increasing the abundance of the consumers over time. In food webs, the increased abundance or biomass of the consumer trophic level, relative to the consumed trophic level is called a trophic cascade. It makes allusion to the appearance in graphs of successive reduced and increased biomass in the trophic levels in the system. These increasing and decreasing consumption rates oscillate through time and ripple through generations and are one cause of population dynamics. An interesting and useful parameter for ecologists is to find out at what relative consumption rates trophic cascades occur, for they cannot occur under elevated food resources (Polis et al., 2000). It has been postulated that the microarthropods, particularly the Collembola and Oribatida, have a more significant role in the fungal pathway (Faber, 1991; Schneider and Maraun, 2005; Jonas et al., 2007). Several additional papers discussed fungivory by soil microarthropods (e.g., Maraun et al., 2003; Schneider et al., 2004; Pollierer et al., 2009; Thiele-Bruhn et al., 2012), but few tried to quantify rates of hyphae consumption except for individual species, such as Collembola (Jonas et al., 2007), while others focused on feeding preferences (e.g., Ruess et al., 2005; Koukol et al., 2009; Semenina and Tiunov, 2011). However, little is known regarding the quantities of fungal mycelium consumed and assimilated by microarthropods species when in monoculture, in competition, or with predation pressure. Likewise there is little insight into the microarthropods foraging behavior within the soil itself, or their response to the distribution and quality of food resources (Adl, 2003; Hassall et al., 2006). Considering there are 10^1^–10^2^ m of hyphae in one gram of fertile soil (Leake et al., 2003), this is a large biomass contribution to soil nutrient turnover that ought to be studied more. The difficulty has been in designing microcosms with compatible species, and to develop a technique with sufficient sensitivity to work with the small number of individuals in a microcosm experiment.

One of the main methods of differentiating between feeding preferences and measuring consumption rates is the use of stable isotopes (Tiunov, 2007; Semenina and Tiunov, 2011), as this can provide a time-integrated measure of the trophic position of soil animals (Pollierer et al., 2009). An organisms’ tissues exhibit a fixed isotopic enrichment in relation to their diet; therefore stable isotopes can be measured to assess the assimilation rates over the long-term (Peterson and Fry, 1987). Introducing an enriched food source as a pulse provides a mechanism to accurately monitor consumption in a controlled environment (Bradford et al., 2012). The addition of a substrate with a distinct isotopic signature can be traced into newly synthesized compounds and tissues (Elfstrand et al., 2008). The introduction of a food source which is enriched above natural abundance levels with stable isotopes into the soil food web can elucidate feeding interactions as they are happening. In ecology, shifts in ^13^C indicate change in diet, and the ^13^C natural abundance value indicates an equilibrium average of the various food sources. In contrast ^15^N values have been used to indicate position in trophic level in a food web – the higher the δ ^15^N value, the higher the trophic level. It also shows categorically that the food source has been consumed and assimilated.

However, Maraun et al. (2011) advised there was a need to investigate the individual feeding channels, but there is a difficulty differentiating basal resources using stable isotopes at natural abundance. The problem with the interpretation of natural abundance studies with the rare stable isotopes ^13^C and ^15^N is that it provides an equilibrium and average value of all the sources of food consumed (Moore et al., 2004; Schneider et al., 2004; Pollierer et al., 2009; Schneider and Maraun, 2009). These studies indicated there was sometimes poor resolution between some trophic compartments without more detailed studies. To address these concerns with natural abundance field data, it is necessary to turn toward microcosms to make specific measurements. In these microcosm studies, it is important to consider both competition and predation and not just single species consumption rates, as single species results might not be representative of field data (Wardle and Yeates, 1993). A number of studies employing stable isotopes focused on quantifying the bacterial energy pathway (e.g., Murray et al., 2009; Crotty et al., 2011b, 2012a), and here this study focuses on the fungal pathway. Tracking the energy flux through the fungal-feeding channel within soil food webs is more complicated than the bacterial channel (Crotty et al., 2012b). Fungi are easily shredded during sampling, and are not easily re-introduced *in situ*, highlighting a need to validate the use of enriched isotope tracers in microcosm settings to study fungal energy pathways.

Energy flux dynamics in multitrophic interactions food webs provide the data to understand biodiversity-ecosystem functioning. Yet, there are few studies portraying this, despite the crucial link these studies would provide in linking trophic interactions with ecosystem function (Barnes et al., 2018). The overall aim of the experiments presented here, were to study the effects of species interactions (competition and predation) on the rate of consumption and assimilation as determined by isotope ratio mass spectrometry (IRMS). To do this the rate of consumption of saprotrophic fungal biomass by soil microarthropods needed to be determined. We tested the hypothesis that mycelium assimilation by the fungivores, measured as body mass enrichment, will be the same for each of the species used. We tested the hypothesis that in the presence of a predator, there is evidence of a trophic cascade, so that consumption of the mycelium is reduced. We also tested the hypothesis that the δ^13^C and δ^15^N isotopic signatures of the predator would be affected by its prey’s isotopic composition.

## Materials and Methods

### General Methods

Soil cores were obtained from a wooded area of Point Pleasant Park in Halifax, Nova Scotia, Canada (latitude 44.621876277, longitude -63.5711053). The microarthropods were extracted from the soil using a Tullgren funnel heat extraction (5 mm mesh) into prepared microcosms. The microcosms were plastic vessels (54 mm diameter, 60 mm height) with a ∼10 mm layer of plaster of Paris mixed with activated charcoal (5:1 by weight, 2 parts powder to 1 part water). Numerous species were found but only some were successfully cultured on fungal mycelia or baker’s yeast in the necessary quantities for the microcosm experiments. Laboratory cultures were established of *Lepidocyrtus curvicollis* (Collembola: Entomobryidae), *Oribatula tibialis* (Acari: Oribatida: Oribatulidae), *Tyrophagus putrescentiae* (Acari: Astigmata: Acaridae), and *Hypoaspis acquilifer* (Acari: Mesostigmata: Laelapidae). Microarthropods were grown on a mixed diet of baker’s yeast (added every few days) and ground dried nettle as organic matter (added weekly), in darkness at room temperature (about 24–26°C).

Fungal cultures were obtained from the same soil as the microarthropods. Saprotrophic fungi were initially isolated by placing peds of soil on potato dextrose agar (1.5%) with Rose Bengal (0.1%) to suppress bacterial contamination. Sub-cultures of the hyphae, which grew out of the soil, were taken in a series to obtain single fungal species in culture that was identified as an ascomycete by microscopy. The minimal medium was composed of 7 g Na_2_HPO_4_, 3 g KH_2_PO, 0.12 g MgSO_4_, 0.011 g CaCl_2_, 0.5 g NaCl_2_ per liter deionised water, with 1.2% w/v technical agar (number 3), which were combined and sterilized (autoclaved at 121°C for 15 min), before the addition of 2.5 and 1 g, respectively, of filter sterilized ^13^C-glucose (C_6_H_12_O_6_) and ^15^N-ammonium chloride (NH_4_Cl) until the agar substrate was enriched to 99.9% (Crotty et al., 2011a). Reference control cultures were also grown prepared in the same way but the glucose and ammonium chloride were at natural abundance of C and N isotopes. Nitrocellulose disks (45 mm dia. Bio-Rad, Ontario, Canada) were placed on top of the agar and inoculated with the mycelium. Fungal hyphae grow without penetrating the agar to ease the transfer of mycelium to microcosms.

The feeding experiments were conducted in Petri dish (diameter 6 cm) with a <5 mm layer of plaster of Paris and activated charcoal at the bottom. In preparation for each experiment, microarthropods were transferred from culture to a new Petri dish without food, and starved for 48 h before the feeding experiments commenced. Fungal biomass was harvested by lifting the nitrocellulose disks from the agar, and each placed in the center of a fresh Petri dish microcosms. Each microcosm received about 20 mg wet weight of mycelium (weight measured and recorded for stable isotope analysis). We verified in controls the fungal mycelium transferred to the experiment Petri plate microcosms did not grow, as there was no substrate.

In preliminary trials to determine optimal experimental conditions, we established that 20 mg of mycelium in the Petri dish area (approximately a quarter of the nitrocellulose disk) could maintain a stable environment for about 20 *Lepidocyrtus* or *Tyrophagus*, with up to 10 *Oribatula;* more than 10 individuals of the predator *Hypoaspis* was too crowded in one dish, especially without prey, but 5–10 with prey provided stable microcosms. *Oribatula* was slow-growing on the cultured fungus, and this species clearly was not a preferred food source. We kept this species so as to provide a range of grazing rates for the competition and predation experiments. These preliminary growth experiments on labeled and un-labeled mycelium were conducted for up to 21 days to establish optimal durations. We established that optimal multispecies microcosm incubations were 5–7 days; shorter incubations (<5 days) showed more variability and lower enrichment; longer periods of up to 2–3 weeks allow recycling nutrients by coprophagy, losing too many prey to the predator, and over-grazing the mycelium. We calculated the amount of consumption using the Petersen and Luxton (1982) equation, Assimilation = consumption – feces.

The experiments were terminated by removing the microarthropods from each dish, grouped by species, killed by submersion in 100% ethanol for <1 min, and samples were processed immediately for analysis by IRMS. The microarthropod filled capsules were dried at 65°C for 48 h, re-weighed, and ^13^C/^12^C and ^15^N/^14^N ratios measured using Costech ECS4040 elemental analyzer coupled to a Delta V Advantage mass spectrometer (Thermo Fisher Scientific, Finnegan, Germany) with a Conflo IV interface (Thermo Fisher Scientific, Finnegan, Germany), following the methods described in Crotty et al. (2013) for low mass samples.

The fecal pellets and exoskeletons remaining in the microcosms were also collected, counted, added to pre-weighed tin capsules, dried at 65°C for 48 h, re-weighed, and ^13^C/^12^C and ^15^N/^14^N ratios measured as described above. The amount of fungi consumed by the microarthropods over the incubation period was calculated and expressed as dry weight per individual per day.

### Competition Experiments With and Without a Predator

This experiment focused on the effect competition or predation had on feeding rates, using the level of isotope enrichment acquired by each species as a proxy for consumption. Stable isotope enriched mycelium was added to ten different treatments (each treatment replicated five times) in microcosms. The treatments were, four single species monocultures (*Lepidocyrtus, Tyrophagus, Oribatula*, and *Hypoaspis*); one with two fungivores in competition (*Lepidocyrtus* and *Oribatula*); one with a fungivore and a predator (*Lepidocyrtus* and *Hypoaspis*); one with three fungivores (*Lepidocyrtus, Tyrophagus*, and *Oribatula*); two microcosm treatments with two fungivores and a predator (either *Lepidocyrtus, Oribatula* and *Hypoaspis* or *Lepidocyrtus, Tyrophagus*, and *Hypoaspis*); and the final microcosm treatment which had all faunal species in competition / predation with each other (*Lepidocyrtus, Tyrophagus, Oribatula*, and *Hypoaspis*) Microcosms were incubated for 7 days, as established above. The number of individuals at the end of each treatment were counted. Following incubation, the Petri dishes were prepared for analysis by mass spectrometry as described above. Individuals of the same species in the same microcosm were bulked due to obtain sufficient biomass for analysis.

### Statistical Analysis

Microarthropod effects on mycelium consumption, fecal pellet biomass and isotopic composition where assessed by a general analysis of variance. Where applicable multiple comparison within tables of means were made using the Student-Newman-Keuls test (Sokal and Rohlf, 1995), with the experiment-wise type-1 error rate set at 5%. In order for the mycelium samples to be within the enrichment thresholds of the mass spectrometer a companion reference sample was used to dilute the isotope signal, consequently the isotopic results were back-calculated using the equations of Hauck and Bremner (1976) to ascertain original enrichment levels. All data were analyzed using GenStat (14th Edition, Payne et al., 2011) and are presented as mean ± standard error, or SED (standard error of the difference), unless otherwise stated.

## Results

### Consumption of Mycelium by Microarthropods

The mean ingestion (consumption rate) and fecal pellet excretion rates over a 21 day single microarthropod incubation with unlabelled mycelium and with ^13^C and ^15^N enriched mycelium (except without *Oribatula* as they were too few) were compared (Table 1). There were no significant differences in microarthropod weight when comparing unlabelled mycelium with highly enriched mycelium between the two experiments after 21 days (*P* = 0.073). There were no significant differences between the consumption rate of *Lepidocyrtus, Oribatula*, and *Tyrophagus* (*P* = 0.352). The weight of each fecal pellet produced per organism was: *Oribatula* (0.04 ± 0.012 μg), *Tyrophagus* (0.33 ± 0.032 μg), and for *Lepidocyrtus* (0.10 ± 0.020 μg). There were no significant differences in the fecal pellet weight excreted per organism per day in the isotopically enriched cultures compared to the natural abundance measurements (*P* = 0.15). There was a significant difference between the fungivorous organisms, with *Tyrophagus* excreting significantly greater quantities than *Lepidocyrtus* and *Oribatula* (*P* = 0.011). The feces of both species showed a lower level of enrichment in ^13^C than the mycelium at the end of the experiment (*Lepidocyrtus APE* 3.23 ( ± 0.506) *P* = 0.033; *Tyrophagus APE* 2.15 ( ± 0.475) *P* = 0.010) compared to mycelium *APE* 5.08 ( ± 0.5735), although not in *APE* ^15^N [*Lepidocyrtus APE* 18.54 ( ± 2.780) *P* = 0.375; *Tyrophagus APE* 23.99 ( ± 3.054) *P* = 0.438] compared to mycelium *APE* 22.15 ( ± 3.449). These results taken together (Table 1) indicate that the three species of fungivores do not assimilate the same amount of the mycelium consumed, and that differences accumulate over time between the enrichment of fecal pellets and body mass among the fungivores.

**Table 1:** Weight of microarthropods and mycelium consumed in daily dry-weight per individual (*n* = 5).

	*Hypoaspis*	*Oribatula*	*Tyrophagus*	*Lepidocyrtus*	Probability (SED)
Weight (μg)	18.87 ( ± 1.017)^a^	1.76 ( ± 0.128)^b^	1.77 ( ± 0.125)^b^	3.16 ( ± 0.105)^b^	*P* < 0.001 (0.734)
Ingested (μg day^-1^)	–0.82 ( ± 6.461)^a^	5.29 ( ± 1.370)^b^	5.11 ( ± 1.105)^b^	4.70 ( ± 1.279)^b^	*P* < 0.013 (3.77)
Excreted (μg day^-1^)	0.00	1.65 ( ± 0.012)^a^	0.58 ( ± 0.032)^b^	0.74 ( ± 0.020)^b^	*P* < 0.011 (0.706)
Excreted/ingested %	**—**	31.2%	11.4%	15.8%	
Body weight ingested (day^-1^)	–0.25 ( ± 0.252)^a^	3.12 ( ± 7.93)^b^	3.10 ( ± 9.01)^b^	1.51 ( ± 4.39)^ab^	*P* < 0.006 (0.921)

*General analysis of variance was performed, where fauna were significantly different a Student-Newman-Keuls test was also performed to find significant differences between groups; where there are significant differences between faunal groups along rows, different letters indicate significance.*

The mesostigmatid predator *Hypoaspis* on its own with mycelium but without prey lost weight and did not excrete fecal pellets. The three fungivorous microarthropods consumed mycelium at about the same rate ∼5 μg individual^-1^ day^-1^, and the differences were not statistically significant. The oribatid *Oribatula* was the least efficient on this fungus mycelium, excreting ∼31% of the ingested fungus while consuming more than three times its body-weight daily. The astigmatid *Tyrophagus* was the most efficient, excreting only about 11½% of the ingested mycelium, but it also consumed about three times its body-weight. The collembolan ingested about 1½ times its body weight daily and excreted about 16% of the ingested mycelium. *Lepidocyrtus* was the only organism to produce exoskeletons in enough numbers to collect and weigh. Each collembolan exoskeleton weighed 1.10 μg (± 0.244) and equated to each individual collembolan shedding its exoskeleton 1.17 (± 0.096) times within a 21 day period.

Analyzing the organisms, their fecal pellets, and the mycelium that remained at the end of the incubations, there were no significant differences between the mycelium ^13^C and ^15^N atom% levels and those in the body mass of *Lepidocyrtus, Oribatula*, or *Tyrophagus P* > 0.1 for all fungivores.

### Survival

In competition experiments with and without the predator, the number of surviving individuals at the end of the incubation in each microcosm is informative (Table 2). Individuals are lost to predation and poor adaptation to the incubation conditions. The predator *Hypoaspis* survival was about 75% in monoculture without prey. Over half of the *Lepidocyrtus* and *Tyrophagus* survived in monoculture on the mycelium. Surprisingly, even though the mycelium did not provide a good food source to *Oribatula*, there was only a 12% loss of individuals in monoculture (Table 2). The highest survival rates of predators was when there were two or more prey species, although only some combinations are statistically significant. The collembolan *Lepidocyrtus* did not ameliorate survival of the predator. The *Oribatula* losses were greatest when competing for the mycelium with *Lepidocyrtus*, with indiscernible predation effect. *Tyrophagus* losses were least with all three fungivores present and the predator. The *Lepidocyrtus* microcosms show a distinct increase in losses in the presence of the predator, but this observation is nuanced by the observation (above) that the collembolan did not improve survival of the predator. Visually, at the microscope, collembolan were better at escaping, and it was more effort for the predator to capture one.

**Table 2:** Loss (%) of individuals in each treatment.

Treatment	*Lepidocyrtus*	*Oribatula*	*Tyrophagus*	*Hypoaspis*
Monoculture	38 ( ± 9.8)^abc^	12 ( ± 5.8)^a^	46 ( ± 11.0)	24 ( ± 2.4)^ab^
Two competitors	28 ( ± 6.2)^ab^	44 ( ± 6.8)^ab^	–	–
Three competitors	18 ( ± 8.0)^a^	18 ( ± 4.9)^ab^	41 ( ± 6.8)	–
One fungivore and predator	67 ( ± 7.7)^d^	–	–	32 ( ± 8.0)^b^
Two competitors and predator	68 ( ± 10.7)^cd^	–	48 ( ± 14.9)	6 ( ± 6.0)^a^
Two competitors and predator	56 ( ± 9.3)^bcd^	42 ( ± 8.6)^b^	–	16 ( ± 8.7)^ab^
Three competitors and predator	71 ( ± 5.1)^cd^	48 ( ± 10.2)^b^	13 ( ± 6.0)	2 ( ± 2.0)^a^
Probability	*P* < 0.001	*P* = 0.017	*P* = 0.183	*P* = 0.011
SED	11.46	11.51	16.68	8.12

*Numbers indicate mean ± SE (*n* = 5) % loss of organisms at the end of competition/predation experiments. General analysis of variance found fauna to have significantly different losses dependent on species (*P* < 0.001); general ANOVA was performed, on individual faunal groups to assess differences between treatments (monoculture, competition with or without predator). Student Newman-Keuls test was also performed to define where the significant differences where between treatments; where there are significant differences between treatments within faunal groups different letters indicate significance.*

### Species Interactions Effect on Consumption

**FIGURE 1:**
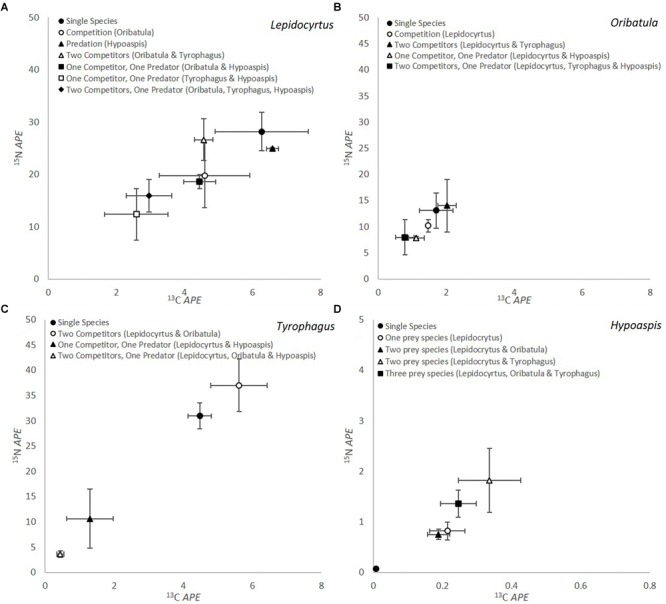
Isotopic label of microarthropods through consumption of fungal mycelium for **(A)** *Lepidocyrtus* (Collembola), **(B)** *Oribatula* (Oribatida), **(C)** *Tyrophagus* (Astigmata), and **(D)** *Hypoaspis* (Mesostigmata) in microcosms with different combination of competitors with/out predators (*n* = 5). The *Hypoaspis* graph **(D)** is at a different scale from the others. The microarthropod ^13^C and ^15^N value at the beginning of each experiment is at the 0 mark of both axis, and we plot the enrichment transferred to the microarthropods in atom percent enrichment (APE). It does not represent the natural abundance value in nature, as these organisms were cultivated in the laboratory prior to the feeding experiment.

The results of the competition-predation microcosm incubation experiments with stable isotope enrichment are summarized in Figure 1. For all these graphs the microarthropod ^13^C and ^15^N value at the beginning of each experiment is at the 0 mark of both axis, and we plot the enrichment transferred to the microarthropods in atom percent enrichment (APE). It does not represent the natural abundance value in nature, as these organisms were cultivated in the laboratory for about 2 years.

*Lepidocyrtus* (Figure 1A) consumption of mycelium differed significantly depending on competition/predation for both isotopes (*APE* ^13^C *P* = 0.010 and *APE* ^15^N *P* = 0.018). Consumption was numerically greatest when incubated as a single microcosm. When *Lepidocyrtus* was incubated solely with the predator *Hypoaspis* (black triangle, Figure 1A) there was very little change in ^13^C or ^15^N value, indicating consumption did not decrease significantly, although survival reduced to 33% (Table 2). When in competition with *Oribatula* (open circle, Figure 1A) or when the microcosms contained two competitors, the value is not statistically different than when it was on its own, indicating that *Oribatula* (and *Tyrophagus*) in fact did not pose a competition threat to *Lepidocyrtus*. When *Lepidocyrtus* is incubated with the predator *Hypoaspis* the ^13^C value is significantly reduced (less consumption) but not the ^15^N value (no change in trophic level, and as results above). When both competitors are present (clear triangle), the three fungivores interfere with each other’s grazing so as to reduce the ^15^N value and the ^13^C value compared to *Lepidocyrtus* alone or with *Oribatula*. In the presence of the predator and *Oribatula* as the only competitor, *Lepidocyrtus* (shaded square) values are not statistically different than with *Oribatula* alone, indicating it is relatively unbothered by predation in this combination. In the absence of the predator but both competitors (clear triangle), there is an intermediate amount of grazing. It clearly shows the difference in competition imposed on *Lepidocyrtus* by *Tyrophagus* but not by *Oribatula.* Without *Oribatula* (clear circle), but with competition from *Tyrophagus* and predation from *Hypoaspis* (clear square), the ^13^C value is reduced but not the ^15^N value when compared to *Lepidocyrtus* alone, indicating reduced mycelium consumption. Curiously, when the predator and both competitors are present with *Lepidocyrtus* (shaded diamond), the value overlaps that of competition with *Oribatula* alone (clear circle). This value is statistically the same as *Lepidocyrtus* with the predator alone for ^15^N, and for *Lepidocyrtus* alone for ^13^C. However, when the microcosm included *Tyrophagus* as a competitor and predators (open square, black diamond, Figure 1A), significantly less consumption occurred. At the microscope in these microcosms, *Lepidocyrtus* seemed unbothered or less-so by the predator, which preferred to chase after the two other fungivores, and thus alleviating competition from the two other fungivores. Feces enrichment levels were also measured for all the different treatments containing *Lepidocyrtus* and there were no significant differences in enrichment levels dependent on species interaction (*P* = 0.634 for ^13^C and *P* = 0.498 for ^15^N).

The amount of isotopic enrichment was relatively lower in *Oribatula* (Figure 1B), as this fungus was not a preferred food source for this species. However, there were no significant differences between treatments in enrichment levels for either ^13^C or ^15^N (*P* = 0.194 and *P* = 0.276, respectively) for *Oribatula*. Comparison of enrichment through grazing between *Lepidocyrtus* and *Oribatula* across the different treatments showed significant differences in enrichment levels in both ^13^C and ^15^N (*P* < 0.001 for both).

*Tyrophagus* showed a different pattern of response to grazing competition and predation (Figure 1C). The level of enrichment from the mycelium between *Lepidocyrtus* and *Tyrophagus* were the same (*P* < 0.001 for both isotopes). As a single species microcosm and in the presence of two competitors, the three species microcosm had significantly greater for both ^13^C and ^15^N when compared to *Tyrophagus* with predation (*P* < 0.001 for both). The *APE* ^15^N result was significantly highest when two competitors were present (open circle, Figure 1C), this could be through coprophagy on fecal pellets of one or both competitors. In the presence of a predator and either *Lepidocyrtus* or *Lepidocyrtus* and *Oribatula* as competitors, the amount of grazing significantly decreased and the isotope APE were greatly reduced for both isotopes. In this composition of the organisms (Figure 1C, black/open triangle) grazing on the mycelium is interfered with through effective grazing competition, combined with organisms trying to escape predation, and by the predation itself.

The level of enrichment obtained by the predator *Hypoaspis* was significantly lower than all other species (*P* < 0.001 for both ^13^C and ^15^N) by one order of magnitude. This is to be expected with the predator being one trophic level above the fungi consumers. When *Hypoaspis* was in a single species monoculture, negligible amounts of enrichment occurred for both ^13^C and ^15^N, however, significant quantities of enrichment were obtained when in microcosms with prey species (*P* = 0.007 for ^13^C and *P* < 0.001 for ^15^N) (Figure 1D). There is a trend for greater levels of ^15^N enrichment when in a microcosm with *Tyrophagus* in comparison to *Lepidocyrtus* or *Oribatula* (open triangle / black square).

Comparing the microcosms with all four organisms together (Figure 1) shows the variety of consumption preferences detected between the predator (Mesostigmata) on each of its prey, and of each of the fungivores (Collembola, Astigmata, Oribatida) on the mycelium. The predator (Table 2 and Figure 1D) far preferred the Astigmata *Tyrophagus* as prey, compared to the oribatid *Oribatula*, or the collembolan *Lepidocyrtus*. This is consistent with our results above, and with our visual observations. In the combination with all four species present, *Lepidocyrtus* consumption of mycelium (Figure 1A) was not affected, as the other fungivores were preferred alternative prey for the predator, and *Oribatula* was not an effective competitor for mycelium consumption. *Tyrophagus* was an effective competitor for *Lepidocyrtus* as both consumed the mycelium at the same rate, but it could barely eat (Figure 1C) because of interference from *Lepidocyrtus*, and preferential predation by *Hypoaspis*. In this combination, *Oribatula* was a secondary prey for *Hypoaspis* but non-etheless preyed upon. In addition, it was in competition with both *Lepidocyrtus* and *Tyrophagus* that both preferred the mycelium, which was less palatable to *Oribatula*. Thus, *Oribatula* and *Tyrophagus* were less enriched in this four species combination.

## Discussion

We selected an ascomycete culture that would sustain three fungivorous microarthropods, and one predatory microarthropod that would prey on the fungivores. We quantified rates of hyphae consumption using ^13^C and ^15^N stable isotopes enriched mycelium. We compared microcosms with and without predation, and with or without competition for changes in hyphae consumption and predation rates.

### Precision and Sensitivity

We showed that standard mass spectrometry instruments could perform at much higher sensitivity by modifying the chemistry of the combustion stage in the sample chamber (Crotty et al., 2013). The IRMS results in that paper showed that 1–10 individual microarthropods were sufficient for reliably accurate measurements. The microcosms in this study consisted of 10–60 individuals at the start of treatments. The resolution of the experiments (Figure 1) are the mean values with five replications. The exception was the *Oribatula* (Figure 1B) where low consumption showed the pattern but without statistical validity. However, the spread of the estimate of error in these experiments (Figure 1) are reasonably low for IRMS microcosms.

The objective was to obtain measurements of fungal consumption, assimilation and foraging behavior of each microarthropod when affected by competition and/or predation. We understand that in these microcosms we have a flat surface that does not mimic the soil three dimensional structure. Within the complexity of the soil matrix there would be a diversity of more or less palatable species to choose from, and behavior of escape and competition interference could be different. Conceivably, in the microcosm the *Furca* (jumping organ, unique to Collembola), may have reduced the time needed to avoid predation (Hopkin, 2002) and resume fungal consumption; and in contrast in the soil matrix, Collembola could be harder to find but less able to escape. In addition, the fungivore consumers were fed a single species of fungus continuously that does not represent a more mixed diet in the soil. Our 7 days incubation period (from trials of up to 21 days) was chosen to provide sufficient levels of consumption to occur for stable isotope enrichment in 2nd and 3rd trophic levels, while providing the number of individuals necessary for isotopic analysis.

### Differential Consumption-Predation

In natural abundance stable isotope studies, due to isotope fractionation during metabolism, δ^13^C values indicate the source of food and the amount of consumption while the δ^15^N values indicate the amount consumed and the trophic level of the species (Tiunov, 2007; Heijboer et al., 2018). In environmental samples with natural abundances, the rare stable isotopes ratio to common isotopes (^13^C and ^15^N to ^12^C and ^14^N) reflect equilibrium values of the isotopes from diverse food sources. That is not necessarily the case in microcosms with species in culture and then in experimental treatments, even though the microarthropods were kept on the same mycelium for many months prior to the treatment. The natural abundance ratios of the three fungivores growing on the same mycelium for several months showed variations in isotope levels between the different microarthropods. If all three species had the same diet and consumed the same amount of the mycelium, then *Lepidocyrtus, Oribatula* and *Tyrophagus* would show the same isotope ratios, but that is not the case. The differences are due to probably an array of reasons, such as differences in development stage or exoskeleton - coprophagy / fecal pellet consumption, and selective metabolism (Semenina and Tiunov, 2011; Maraun et al., 2011; Hatch, 2012; Potapov et al., 2014).

The δ^13^C values of each fungivore in monoculture (Figure 1A–C, dark circles) reflects the equilibrium amount and value of δ^13^C when undisturbed. During isotopic enrichment treatment incubation, the rare stable isotope is accumulated and the graphs show enrichment for both isotopes compared to the starting point. However, with increasing disturbance from competition and predation in mixed cultures, the δ^13^C values show less enrichment (^13^C APE) moving closer to the zero value (the equilibrium value at the start).

There was a visible trend that *Tyrophagus* was a preferred prey for *Hypoaspis*, as those microcosms provided a greater amount of enrichment to the predator. It is often stated that oribatida live relatively sheltered from predation due to their size and sclerotisation (Peschel et al., 2006; Schneider and Maraun, 2009) which are reasonable assumptions based on the low predation rate on *Oribatula* (Figure 1B,D) although the greatest losses of *Oribatula* were in the microcosms that contained predators (Table 2). Moreover, *Oribatula tibialis* is known to have a defensive cyanogenic aromatic ester (mandelonitrile hexanoate) which would further explain the low predation rate (Brückner et al., 2017). These experiments provide evidence of differential consumption and differential prey preference, so that in the soil optimal foraging would contribute to the behavior of both the fungivores and predators (Hassall et al., 2006; Schneider and Maraun, 2009).

The natural abundance δ^15^N levels were similar between the predatory mite *Hypoaspis* and two fungivores *Lepidocyrtus* and *Oribatula* before commencement of the competition/predation experiment. However, when *Hypoaspis* was in microcosms with a prey species, enrichment from fungal mycelium was traceable to a third trophic level. This exemplifies the caution that is needed when assessing isotope levels in relation to trophic level. Based on natural abundance values from field data increases of 3.4% (Post, 2002) or 1–2% (McCutchan et al., 2003; Illig et al., 2005) have been promoted as indicators of trophic level differences in food webs across ecosystems (see also Moore et al., 2004). Thus, we cannot ignore that these organisms could have consumed eggs, exoskeletons and fecal pellets, engaged in cannibalism, or even predation on juveniles as alternative sources of food in the microcosms, thus appearing to be at a higher trophic level. Some studies have even found Mesostigmata to act as more than just predators by engaging is some opportunist omnivory (Beaulieu, 2012) which would lower its apparent trophic level in natural abundance data.

The literature is rich with examples of microarthropods having a more mixed diet in the field than typically construed, based on casual laboratory observations and from observations in microcosms (Chahartaghi et al., 2005; Erdmann et al., 2007). Other modes of feeding such as coprophagy using exoskeletons and fecal pellets, on eggs, by cannibalism or predation on juveniles have been observed elsewhere (Ponge, 1991; Briones et al., 1999; Ladygina et al., 2008; Endlweber et al., 2009; Fiera, 2014; Feng et al., 2019). For example, *Lepidocyrtus* has a hemimetabolous lifecycle compared to *Oribatula* and *Tyrophagus* which are holometabolous, so that different levels of fractionation may have occurred (Spence and Rosenheim, 2005). *Tyrophagus* is also known to be somewhat omnivorous (Behan-Pelletier, 1999) and therefore, could have potentially found an alternative food source within the microcosms, consuming eggs or immature hatchlings. Not implying that all species are opportunistically or a little omnivorous, we must nonetheless recognize that not all are as specialized as the literature might suggest. These additional sources of consumption that must be considered would affect trophic level interpretations in field data, as they do in microcosms. Microcosm observations then become useful in identifying these cases, and in quantifying energy fluxes in interactions food webs.

### Top-Down Control and Competition Interference

We have shown the fungivorous microarthropods investigated had a voracious appetite, consuming more than their own body weight per day (Table 1). In our preliminary microcosm runs, with incubation of up to 21 days, to avoid the fungivores grazing down the mycelium we provided sufficient non-growing mycelium so as to avoid bottom-up resource limitation on the microcosm. We observed several parameters of optimal foraging theory to be at play in our resource competition interference and predation food webs. One was the interference by competition for the mycelium by other fungivores (Figure 1, and see section “Species Interactions Effect on Consumption”), another was a differential preference for, consumption and excretion of the consumed mycelium biomass. There were more complicated behavioral effects, such as the mycelium consumption of *Tyrophagus* (Figure 1C) reduced by competition from *Lepidocyrtus* especially in the presence of the predator *Hypoaspis* as *Tyrophagus* was a preferred prey. Last, we speculated that the detailed interpretation of the stable isotope results (Figure 1) suggest a more mixed diet that included fecal pellets, exoskeleton, or even juveniles. Excretion by Mesostigmata is mainly fluid with no fecal pellets being formed (Koehler, 1999), but Collembola and Oribatida fecal pellets are important in soil aggregate formation, as a food source for other soil invertebrates, and sometimes contribute significantly to soil structure (Behan-Pelletier, 2003; Maas et al., 2015).

Although the three-dimensional nature of the soil pore space is dissimilar to the Petri dish microcosms, our results convey the pivotal role fungivores must play in nutrient cycling, when considering the amount of fungal mycelium consumed and excreted over time, as both groups represent major trophic and functional components of the soil environment (Leake et al., 2003; Crowther and A’Bear, 2012). The interactive effects of biotic (microarthropods and mycelium) and abiotic (habitat) are central in resolving the functional and ecological responses of the soil food web and this study provides quantified values for use in global models. However, in the natural environment abiotic factors or resource accessibility may affect populations more than competition. Similar studies by others will provide additional comparative data to better understand the complex soil food web interactions, including quantifying the fungivory pathway in detrital food webs.

Here, we have shown methods to ascertain the levels of consumption, competition and predation within the soil food web, knowledge gaps that have been highlighted historically, (Moore et al., 1988; Polis, 1994) but until now have not been quantified. Our results suggest the importance of trophic cascades, these have been modeled, and observed in low productivity systems (de Ruiter et al., 1995; Moore and De Ruiter, 2000; Moore, 2018) but have not been shown empirically until now. Studies such as this set of microcosm studies described here with stable isotope tracers, provide both the required resolution and microscope observations that allow collection of the necessary data for predictive model simulations to be developed.

## Data Availability

The datasets generated for this study are available on request to the corresponding author.

## Author Contributions

This manuscript was carried out by FC during a postdoctoral year with SA. SA wrote the research proposal, obtained the grant, and the overall design of the experiments. FC obtained the measurements and conducted the analysis and calculations. FC wrote the first version of this manuscript. It has since been rewritten, with additional statistical analysis by SA.

## Conflict of Interest Statement

The authors declare that the research was conducted in the absence of any commercial or financial relationships that could be construed as a potential conflict of interest.
